# Supplementary material for: Sorghum Pan-Genome Explores the Functional Utility for Genomic-Assisted Breeding to Accelerate the Genetic Gain
Source: Front Plant Sci. 2021 Jun 1;12:666342. doi: 10.3389/fpls.2021.666342 (PMC8204017; doi:10.3389/fpls.2021.666342)
Supplement: Supplementary Figure 1 — Whole genome sequence of 176 sorghum accessions mapped iteratively to the updated reference sequence assembly and the unmapped sequence reads were assembly iteratively. The plot represents the size of the sequence assembly gained from respective accessions. [file Data_Sheet_1.PDF]

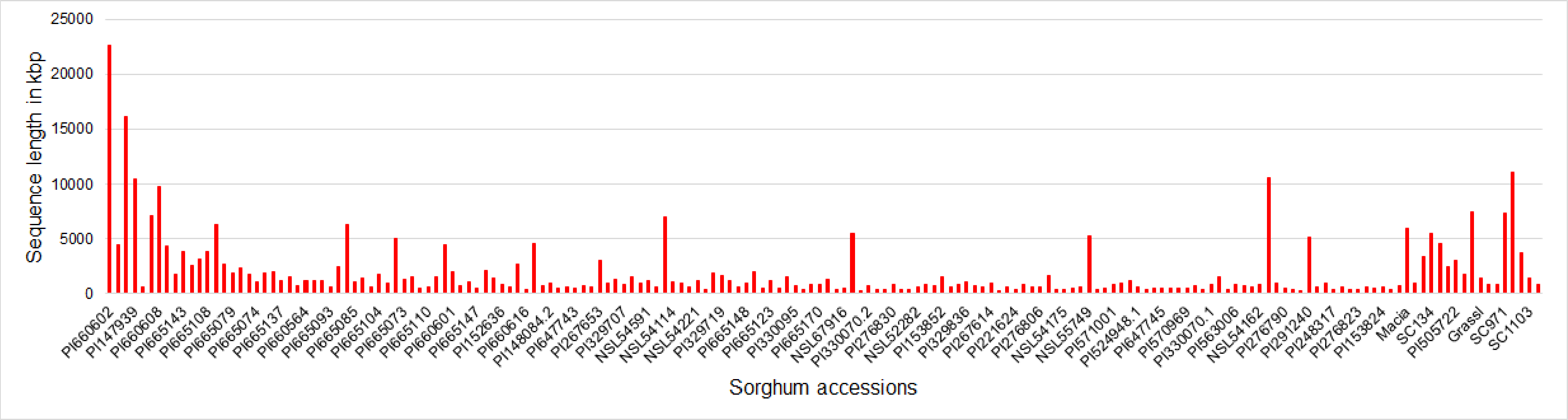

Supplementary Figure 1: Whole genome sequence of 176 sorghum accessions mapped iteratively to the updated reference sequence assembly and the unmapped sequence reads were assembly iteratively. The plot represents the size of the sequence assembly gained from respective accessions.

(A)

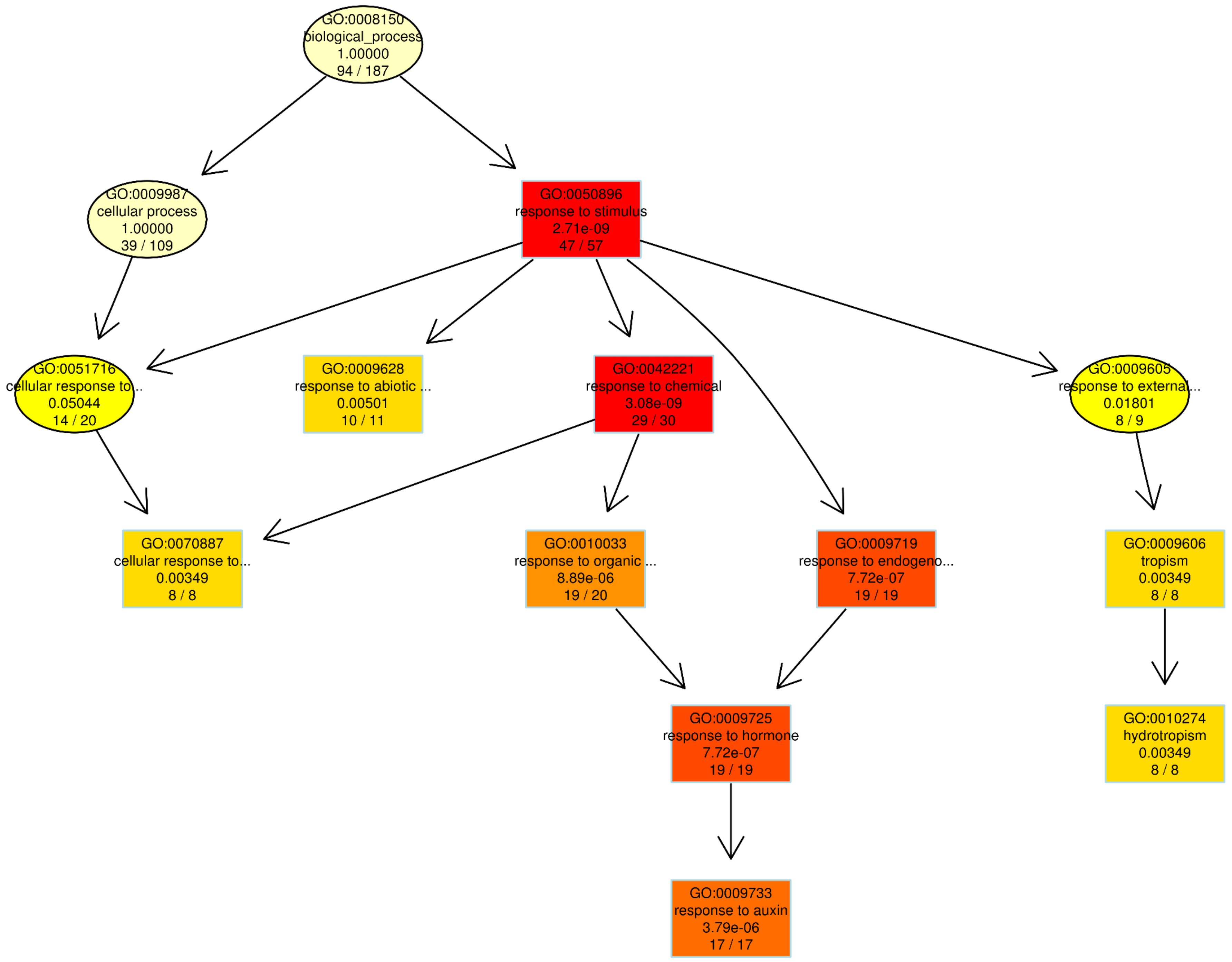
GO:0008150
biological_process
1.00000
94 / 187
GO:0009987
cellular process
1.00000
39 / 109
GO:0050896
response to stimulus
2.71e-09
47 / 57
GO:0051716
cellular response to...
0.05044
14 / 20
GO:0009628
response to abiotic ...
0.00501
10 / 11
GO:0042221
response to chemical
3.08e-09
29 / 30
GO:0009605
response to external...
0.01801
8 / 9
GO:0070887
cellular response to...
0.00349
8 / 8
GO:0010033
response to organic ...
8.89e-06
19 / 20
GO:0009719
response to endogeno...
7.72e-07
19 / 19
GO:0009606
tropism
0.00349
8 / 8
GO:0009725
response to hormone
7.72e-07
19 / 19
GO:0010274
hydrotropism
0.00349
8 / 8
GO:0009733
response to auxin
3.79e-06
17 / 17

(B)

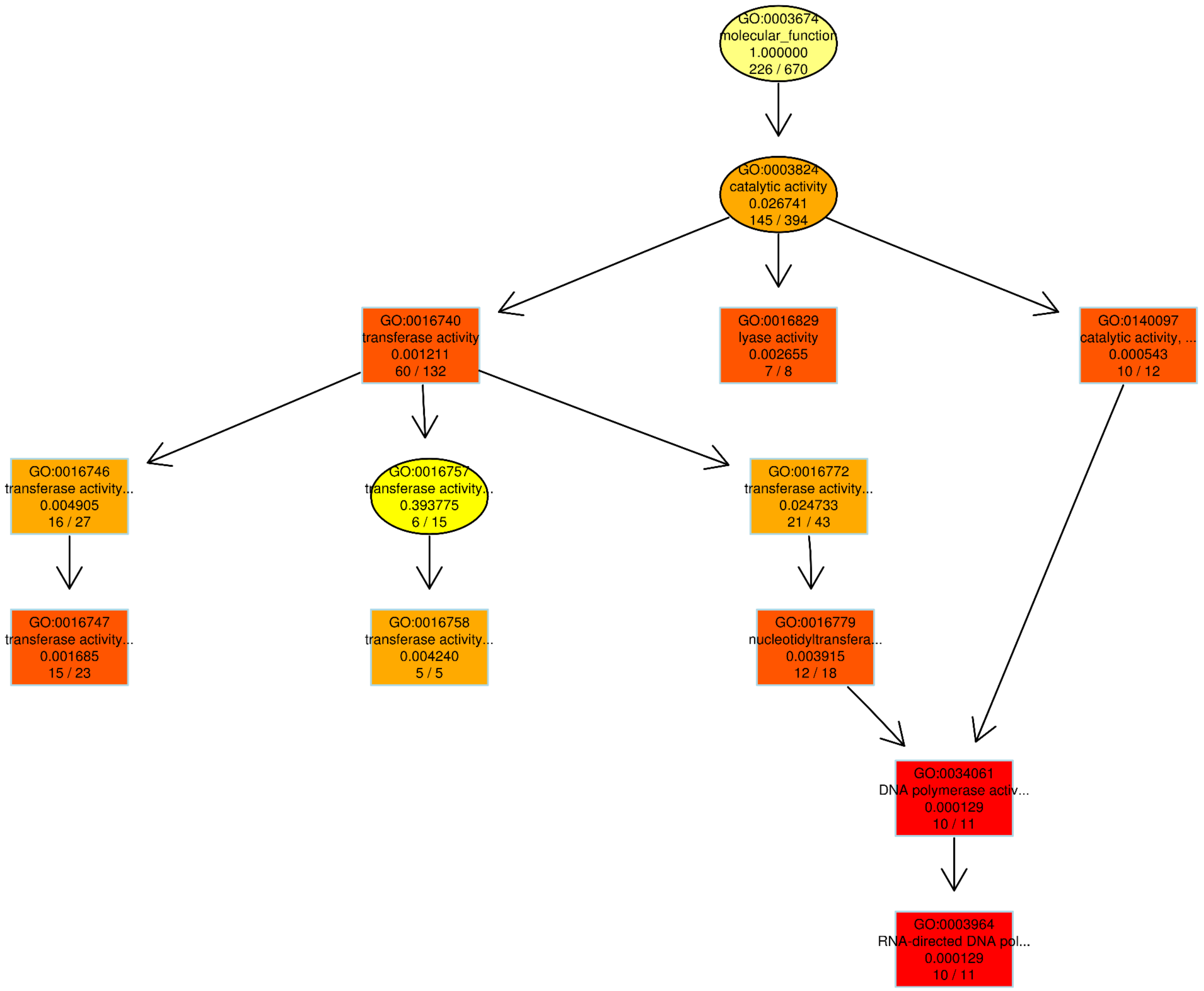
GO:0003674
molecular_function
1.000000
226 / 670
GO:0003824
catalytic activity
0.026741
145 / 394
GO:0016740
transferase activity
0.001211
60 / 132
GO:0016829
lyase activity
0.002655
7 / 8
GO:0140097
catalytic activity, ...
0.000543
10 / 12
GO:0016746
transferase activity...
0.004905
16 / 27
GO:0016757
transferase activity...
0.393775
6 / 15
GO:0016772
transferase activity...
0.024733
21 / 43
GO:0016747
transferase activity...
0.001685
15 / 23
GO:0016758
transferase activity...
0.004240
5 / 5
GO:0016779
nucleotidyltransfera...
0.003915
12 / 18
GO:0034061
DNA polymerase activ...
0.000129
10 / 11
GO:0003964
RNA-directed DNA pol...
0.000129
10 / 11

(C)

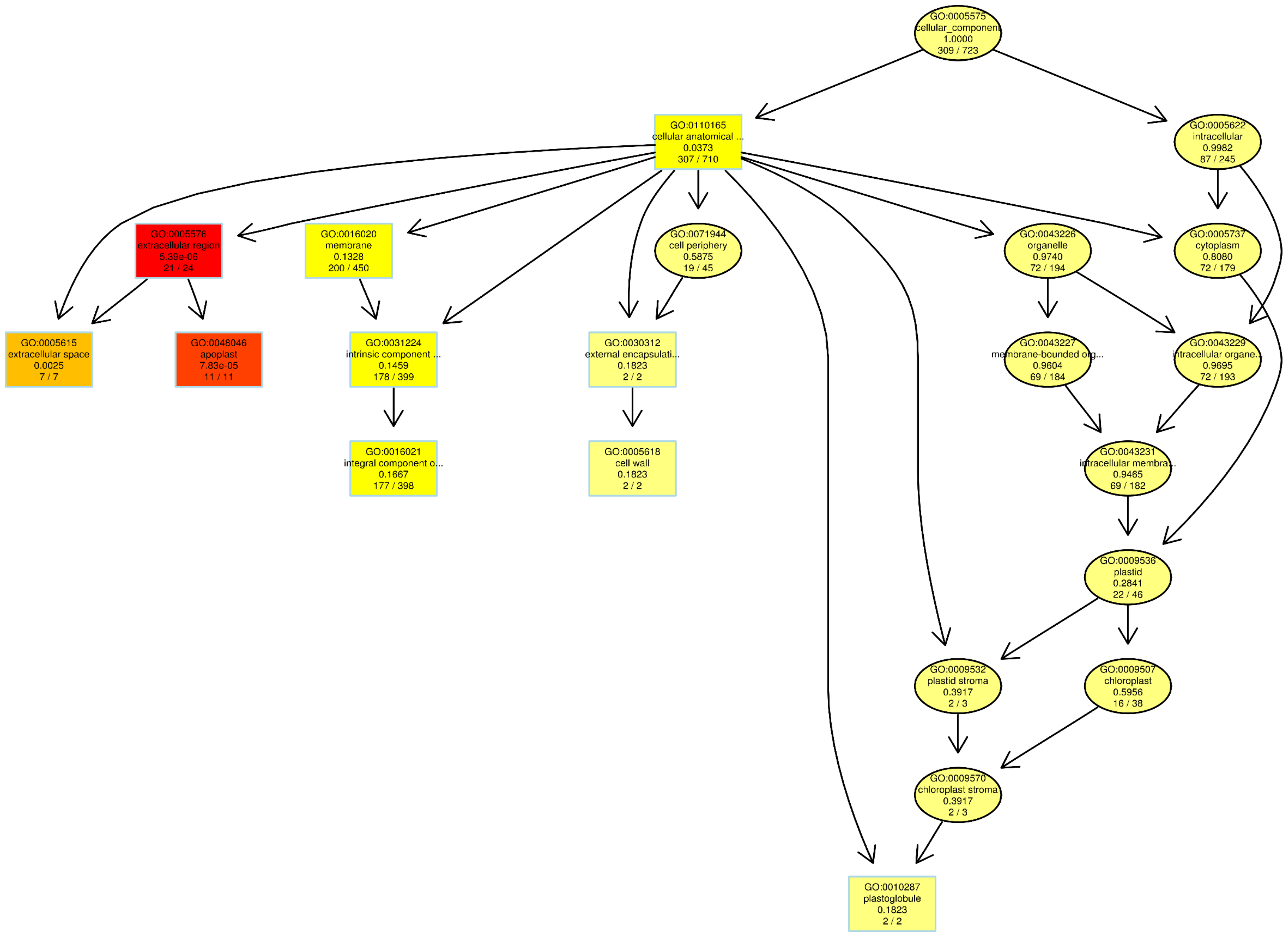

Supplementary Figure 2: The sorghum pangenome variable genes enrichment analysis and the metabolic pathways for **(A)** Biological process **(B)** Molecular function and **(C)** Cellular components. Top 10 GO terms identified for scoring GO terms for enrichment. Rectangles indicate the 10 most significant terms and the colour represents the relative significance ranging from red (most significant) to yellow (least significant). Each node has GO identifier and name with raw p-values and number of significant genes out of total genes annotated.

(A)

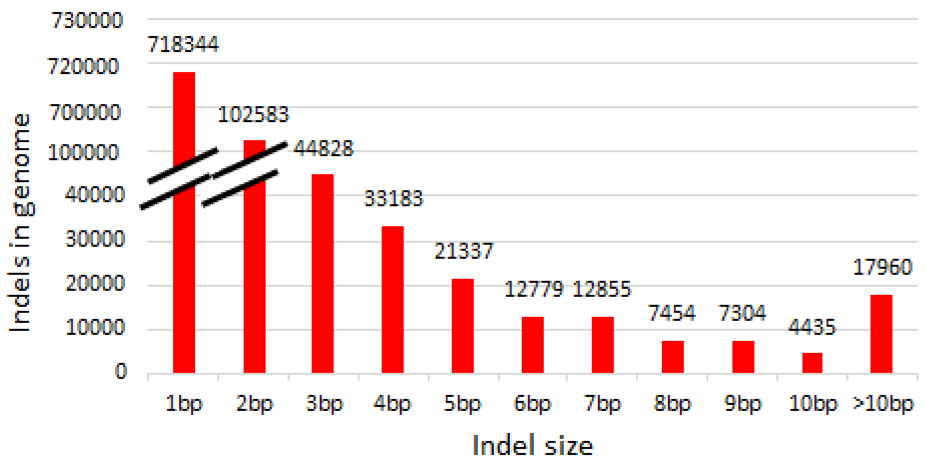

(B)

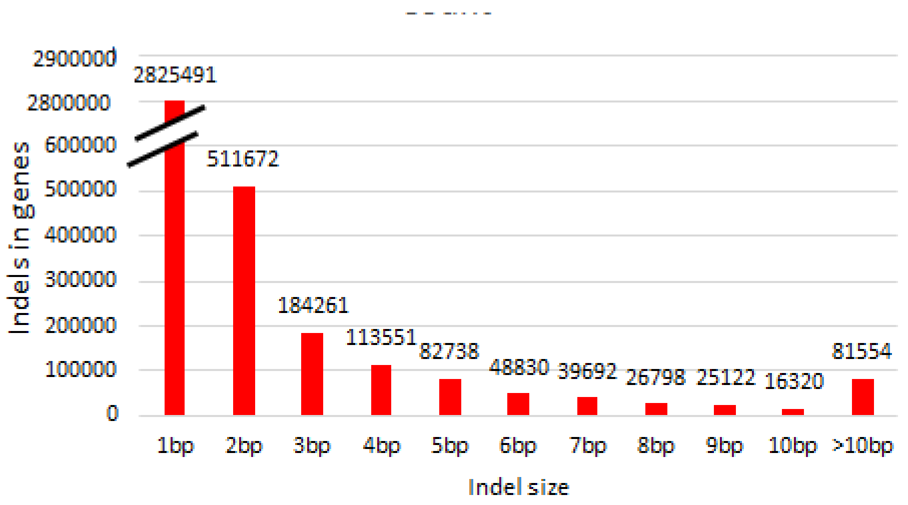

Supplementary Figure 3: Insertions and deletions of various size distribution at sorghum pangenome **(A)** Genome level and **(B)** Gene level

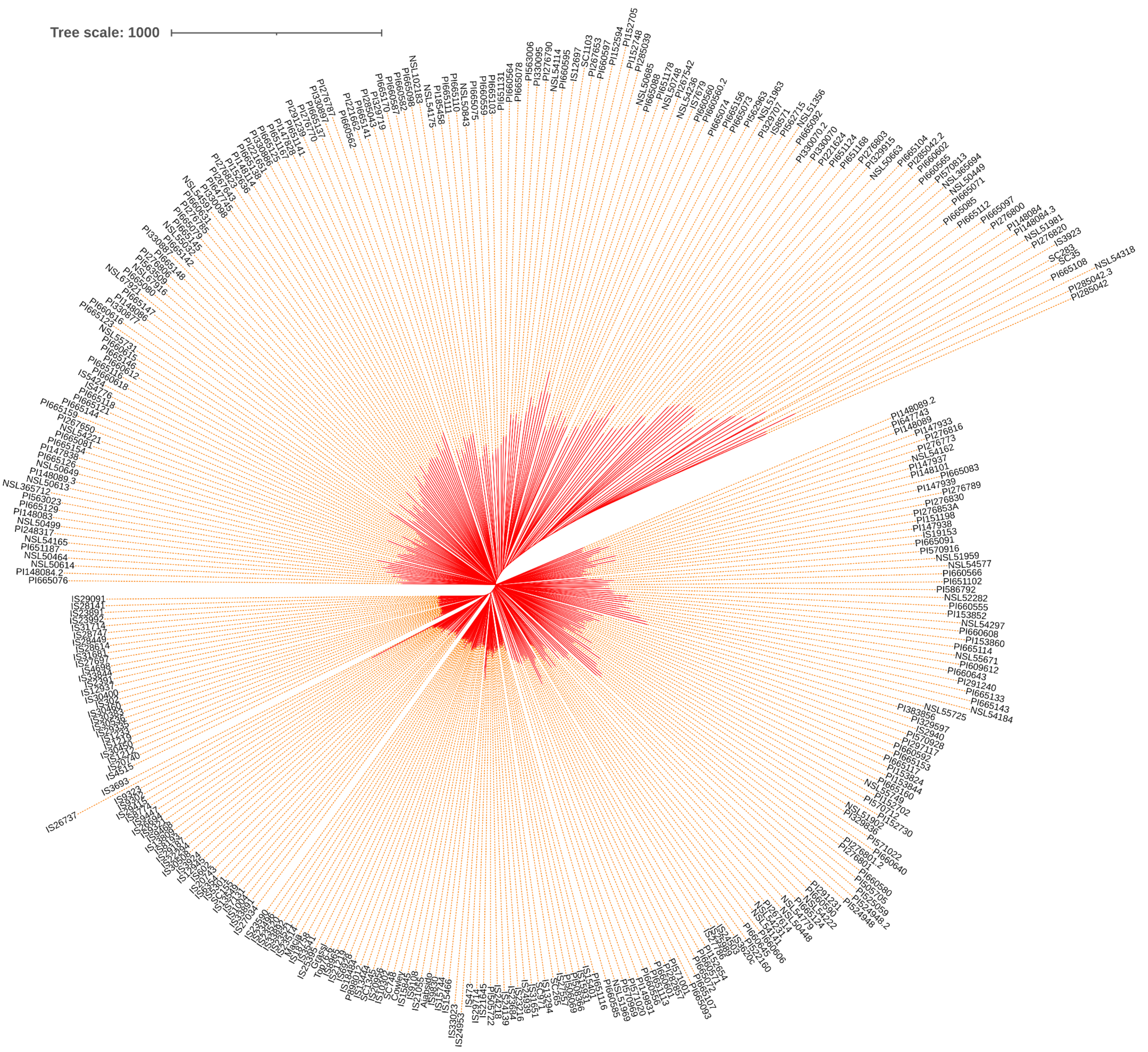

Supplementary Figure 4: The genetic relationship of 354 sorghum accession neighbour joining (with 1000 bootstrap) analysis (the unrooted tree with branch length in red colour).

(A)

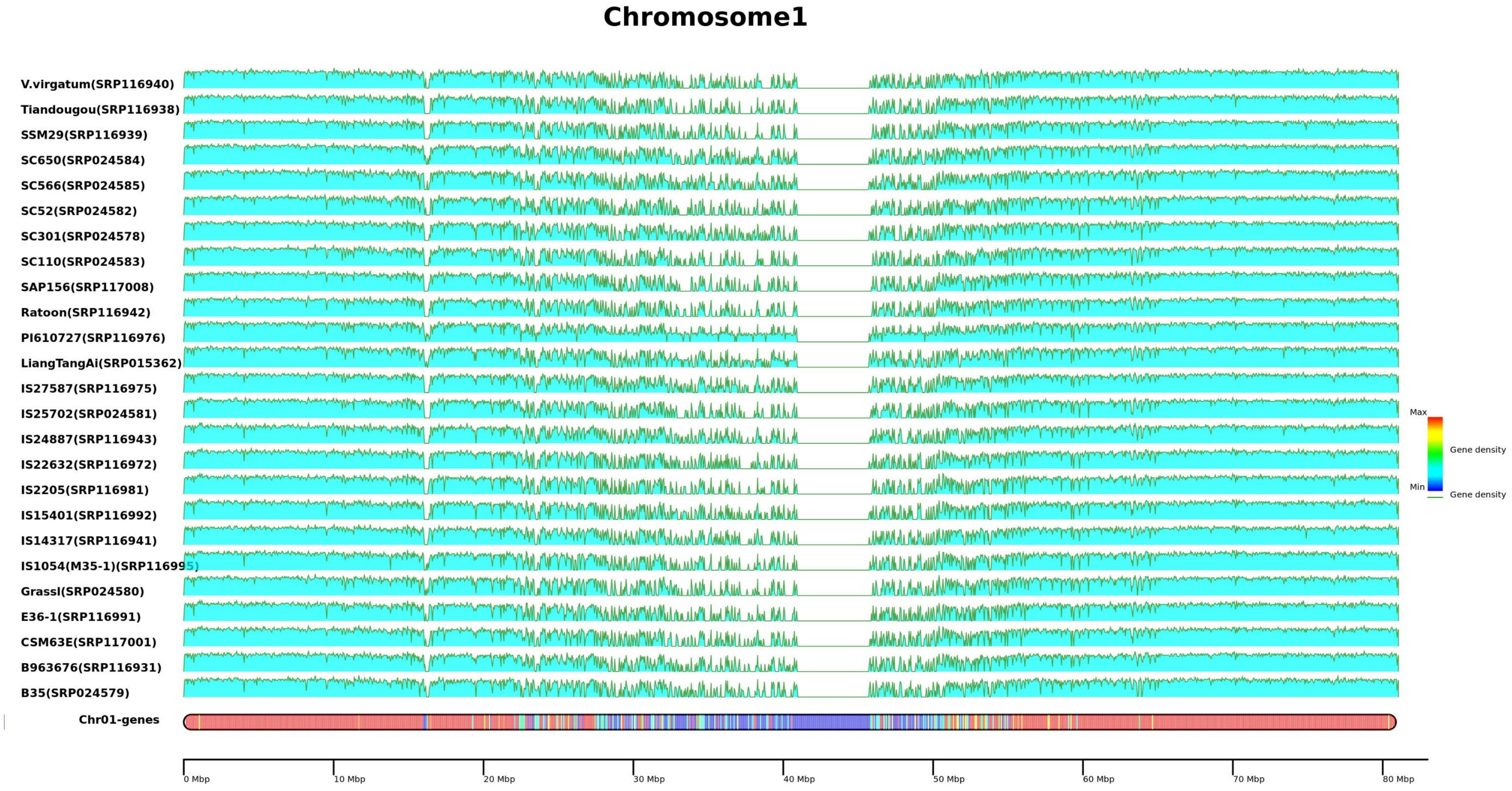

Chromosome1
V.virgatum(SRP116940)
Tiandougou(SRP116938)
SSM29(SRP116939)
SC650(SRP024584)
SC566(SRP024585)
SC52(SRP024582)
SC301(SRP024578)
SC110(SRP024583)
SAP156(SRP117008)
Ratoon(SRP116942)
PI610727(SRP116976)
LiangTangAi(SRP015362)
IS27587(SRP116975)
IS25702(SRP024581)
IS24887(SRP116943)
IS22632(SRP116972)
IS2205(SRP116981)
IS15401(SRP116992)
IS14317(SRP116941)
IS1054(M35-1)(SRP116995)
GrassI(SRP024580)
E36-1(SRP116991)
CSM63E(SRP117001)
B963676(SRP116931)
B35(SRP024579)
Chr01-genes
0 Mbp
10 Mbp
20 Mbp
30 Mbp
40 Mbp
50 Mbp
60 Mbp
70 Mbp
80 Mbp
Max
Gene density
Min
Gene density

(B)

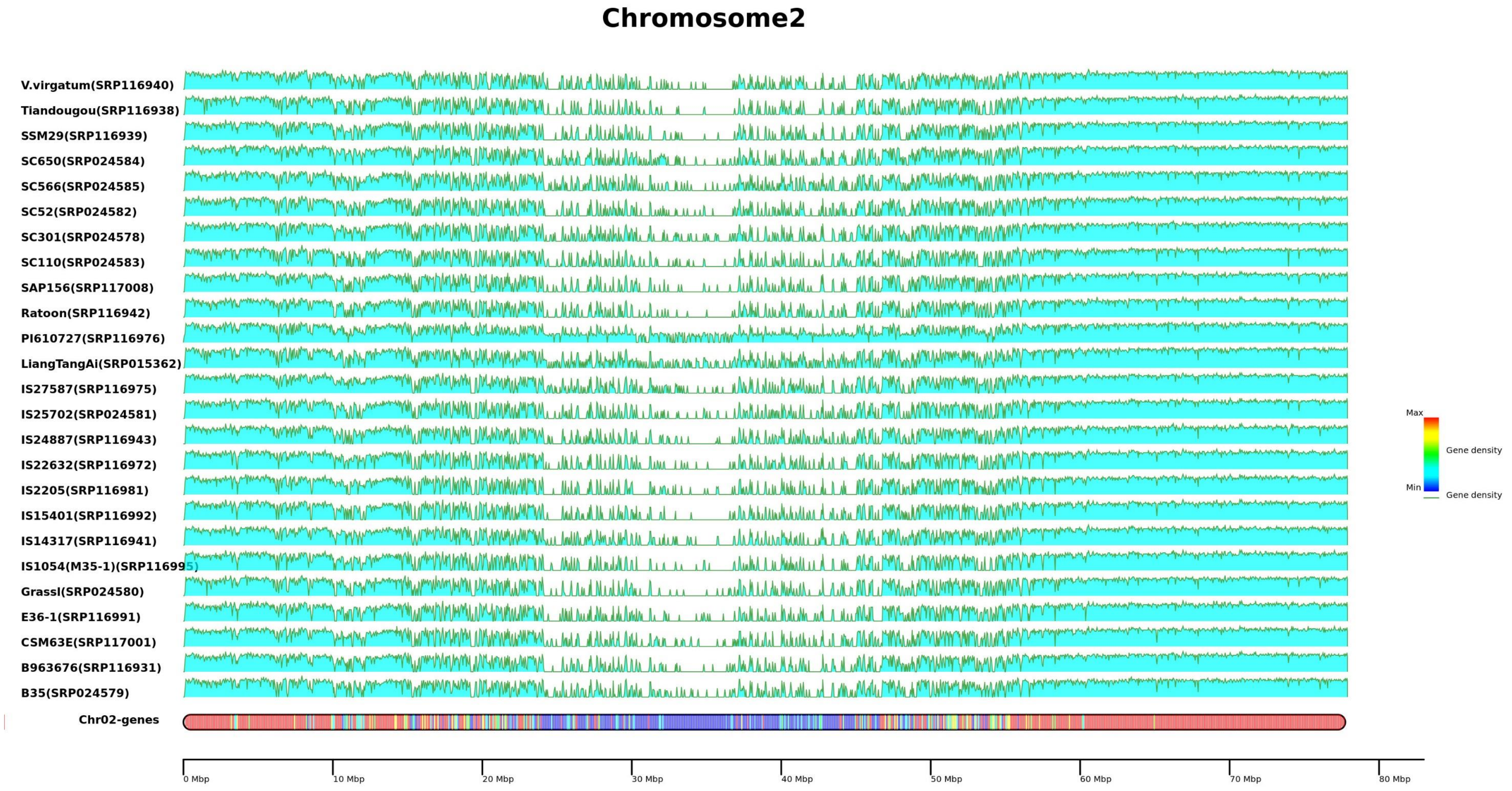
Chromosome2
V.virgatum(SRP116940)
Tiandougou(SRP116938)
SSM29(SRP116939)
SC650(SRP024584)
SC566(SRP024585)
SC52(SRP024582)
SC301(SRP024578)
SC110(SRP024583)
SAP156(SRP117008)
Ratoon(SRP116942)
PI610727(SRP116976)
LiangTangAi(SRP015362)
IS27587(SRP116975)
IS25702(SRP024581)
IS24887(SRP116943)
IS22632(SRP116972)
IS2205(SRP116981)
IS15401(SRP116992)
IS14317(SRP116941)
IS1054(M35-1)(SRP116995)
Grassl(SRP024580)
E36-1(SRP116991)
CSM63E(SRP117001)
B963676(SRP116931)
B35(SRP024579)
Chr02-genes
0 Mbp
10 Mbp
20 Mbp
30 Mbp
40 Mbp
50 Mbp
60 Mbp
70 Mbp
80 Mbp
Max
Min
Gene density
Gene density

(C)

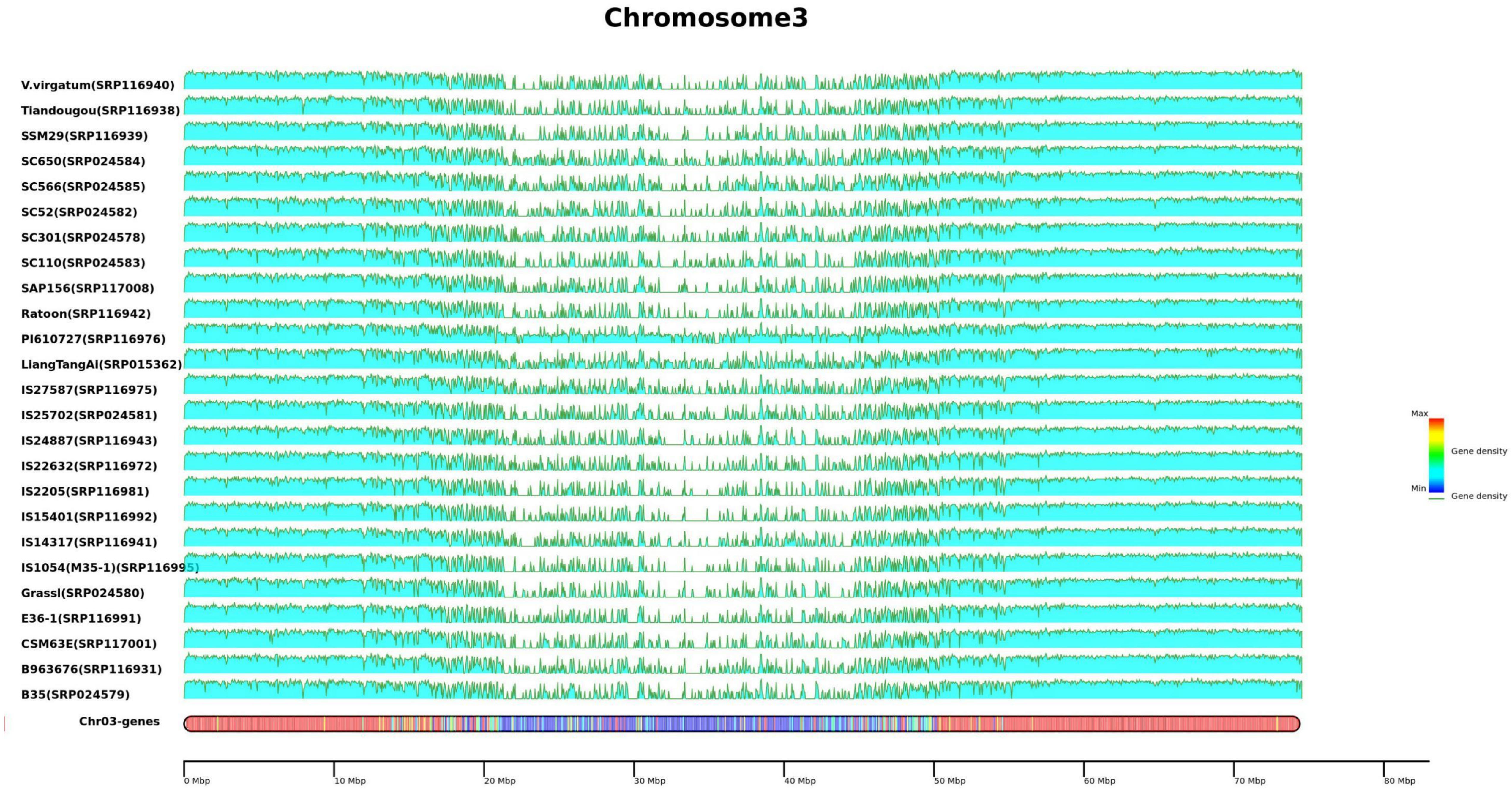
Chromosome3
V.virgatum(SRP116940)
Tiandougou(SRP116938)
SSM29(SRP116939)
SC650(SRP024584)
SC566(SRP024585)
SC52(SRP024582)
SC301(SRP024578)
SC110(SRP024583)
SAP156(SRP117008)
Ratoon(SRP116942)
PI610727(SRP116976)
LiangTangAi(SRP015362)
IS27587(SRP116975)
IS25702(SRP024581)
IS24887(SRP116943)
IS22632(SRP116972)
IS2205(SRP116981)
IS15401(SRP116992)
IS14317(SRP116941)
IS1054(M35-1)(SRP116995)
GrassI(SRP024580)
E36-1(SRP116991)
CSM63E(SRP117001)
B963676(SRP116931)
B35(SRP024579)
Chr03-genes
0 Mbp
10 Mbp
20 Mbp
30 Mbp
40 Mbp
50 Mbp
60 Mbp
70 Mbp
80 Mbp
Max
Min
Gene density
Gene density

(D)

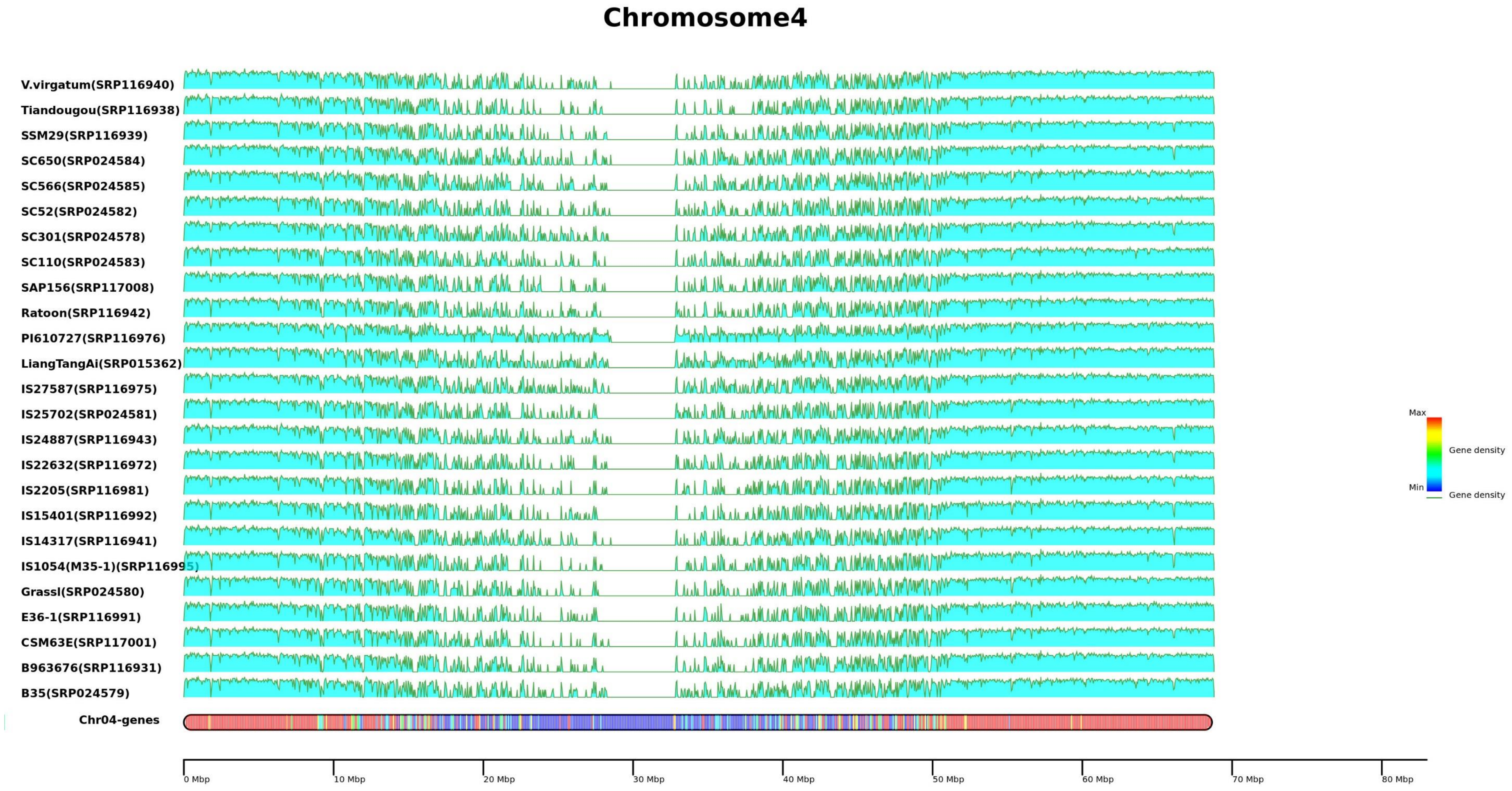

Chromosome4
V.virgatum(SRP116940)
Tiandougou(SRP116938)
SSM29(SRP116939)
SC650(SRP024584)
SC566(SRP024585)
SC52(SRP024582)
SC301(SRP024578)
SC110(SRP024583)
SAP156(SRP117008)
Ratoon(SRP116942)
PI610727(SRP116976)
LiangTangAi(SRP015362)
IS27587(SRP116975)
IS25702(SRP024581)
IS24887(SRP116943)
IS22632(SRP116972)
IS2205(SRP116981)
IS15401(SRP116992)
IS14317(SRP116941)
IS1054(M35-1)(SRP116995)
Grassl(SRP024580)
E36-1(SRP116991)
CSM63E(SRP117001)
B963676(SRP116931)
B35(SRP024579)
Chr04-genes
0 Mbp
10 Mbp
20 Mbp
30 Mbp
40 Mbp
50 Mbp
60 Mbp
70 Mbp
80 Mbp
Max
Min
Gene density
Gene density

(E)

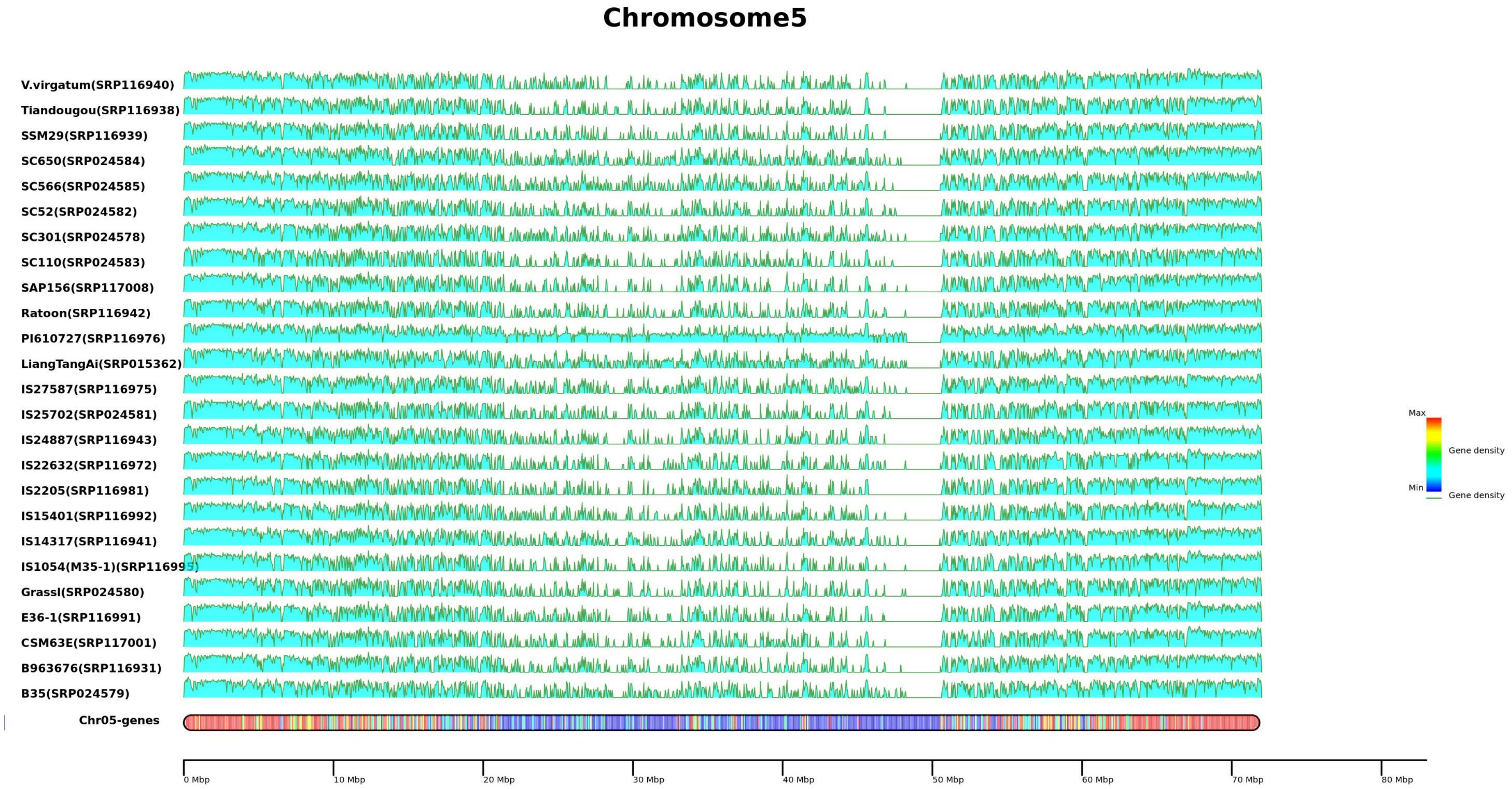
Chromosome5
V.virgatum(SRP116940)
Tiandougou(SRP116938)
SSM29(SRP116939)
SC650(SRP024584)
SC566(SRP024585)
SC52(SRP024582)
SC301(SRP024578)
SC110(SRP024583)
SAP156(SRP117008)
Ratoon(SRP116942)
PI610727(SRP116976)
LiangTangAi(SRP015362)
IS27587(SRP116975)
IS25702(SRP024581)
IS24887(SRP116943)
IS22632(SRP116972)
IS2205(SRP116981)
IS15401(SRP116992)
IS14317(SRP116941)
IS1054(M35-1)(SRP116995)
GrassI(SRP024580)
E36-1(SRP116991)
CSM63E(SRP117001)
B963676(SRP116931)
B35(SRP024579)
Chr05-genes
0 Mbp
10 Mbp
20 Mbp
30 Mbp
40 Mbp
50 Mbp
60 Mbp
70 Mbp
80 Mbp
Max
Min
Gene density
Gene density

(F)

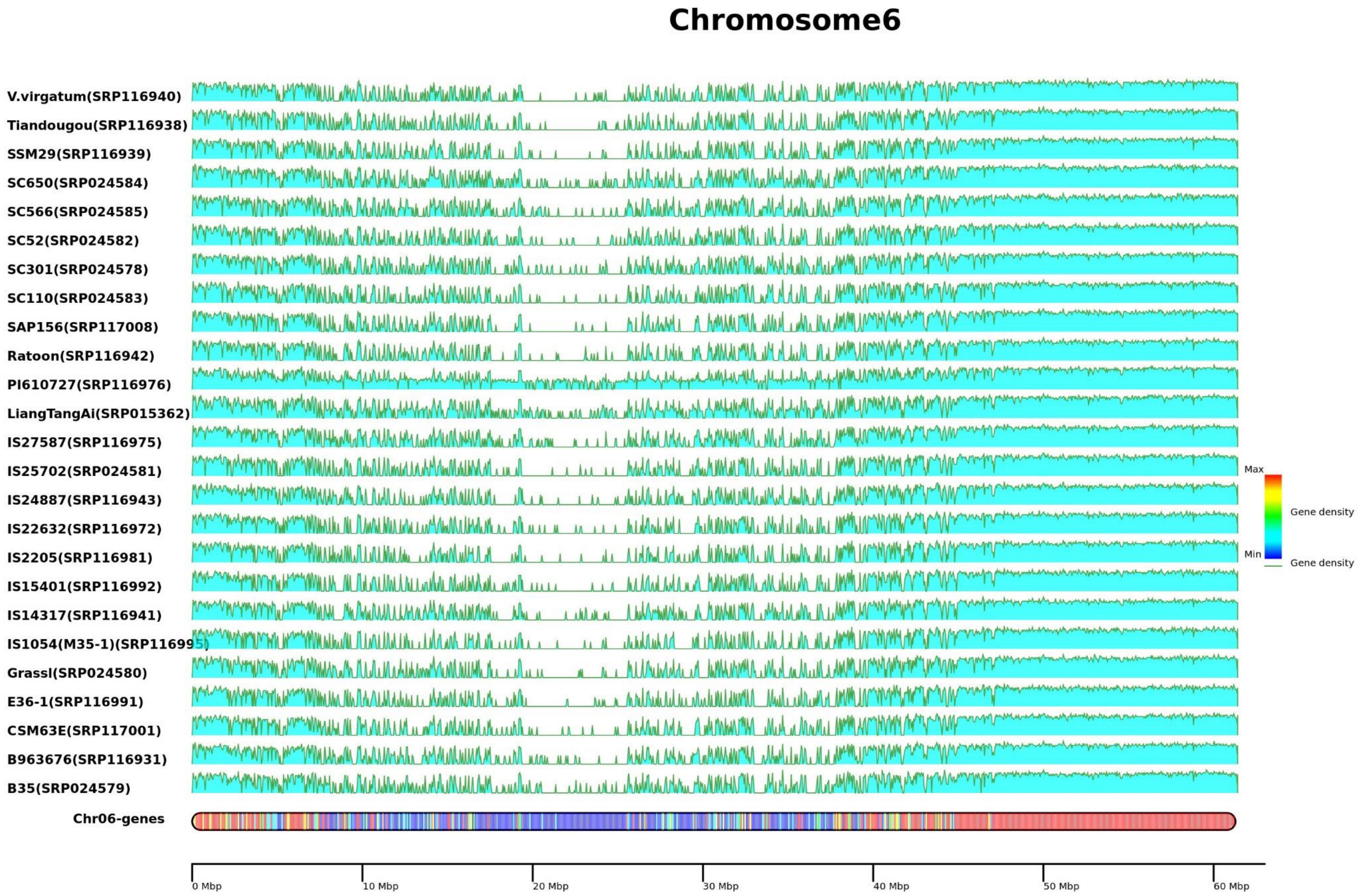

Chromosome6
V.virgatum(SRP116940)
Tiandougou(SRP116938)
SSM29(SRP116939)
SC650(SRP024584)
SC566(SRP024585)
SC52(SRP024582)
SC301(SRP024578)
SC110(SRP024583)
SAP156(SRP117008)
Ratoon(SRP116942)
PI610727(SRP116976)
LiangTangAi(SRP015362)
IS27587(SRP116975)
IS25702(SRP024581)
IS24887(SRP116943)
IS22632(SRP116972)
IS2205(SRP116981)
IS15401(SRP116992)
IS14317(SRP116941)
IS1054(M35-1)(SRP116995)
Grassl(SRP024580)
E36-1(SRP116991)
CSM63E(SRP117001)
B963676(SRP116931)
B35(SRP024579)
Chr06-genes
0 Mbp
10 Mbp
20 Mbp
30 Mbp
40 Mbp
50 Mbp
60 Mbp
Max
Min
Gene density
Gene density

(G)

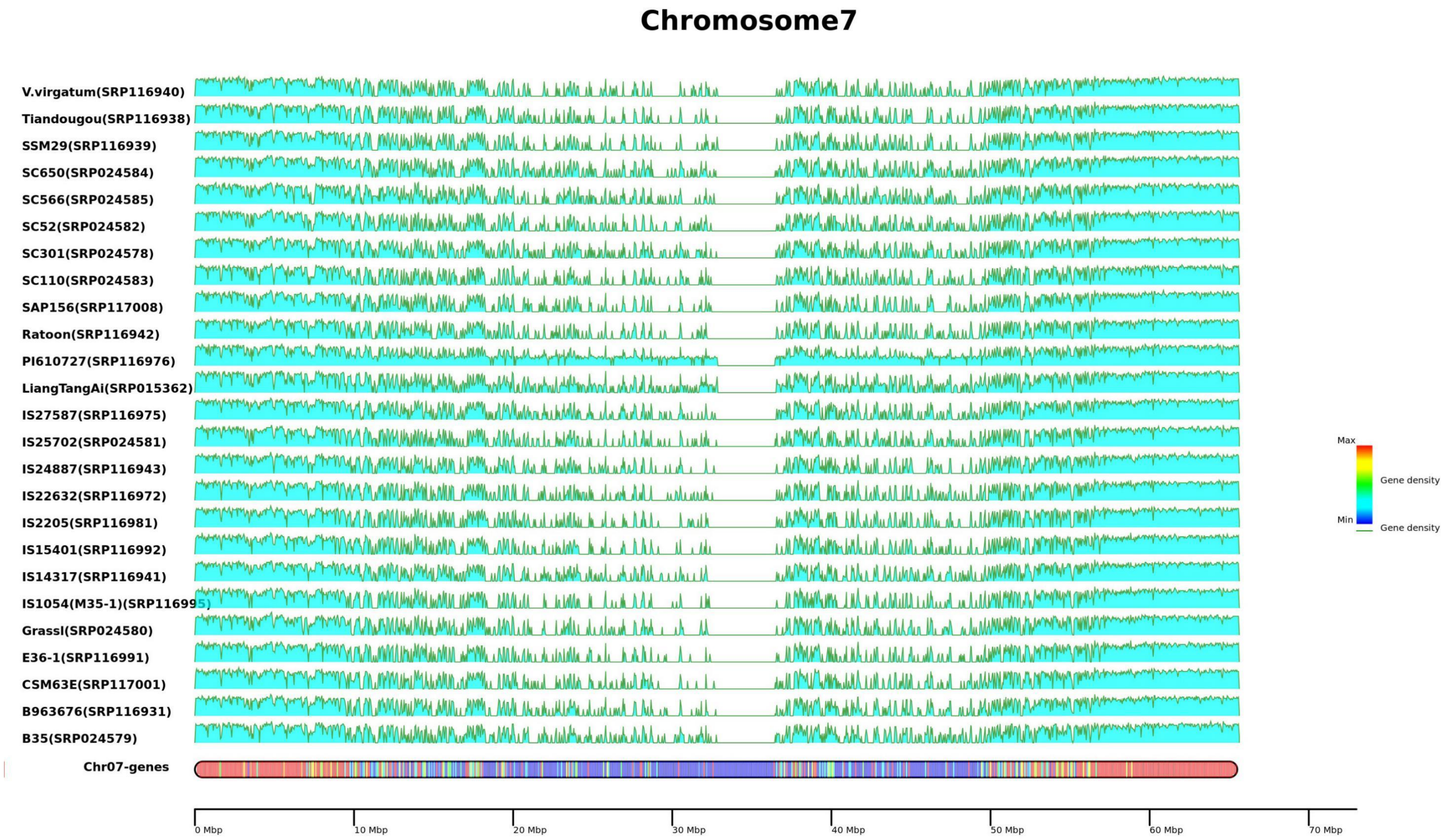
Chromosome7
V.virgatum(SRP116940)
Tiandougou(SRP116938)
SSM29(SRP116939)
SC650(SRP024584)
SC566(SRP024585)
SC52(SRP024582)
SC301(SRP024578)
SC110(SRP024583)
SAP156(SRP117008)
Ratoon(SRP116942)
PI610727(SRP116976)
LiangTangAi(SRP015362)
IS27587(SRP116975)
IS25702(SRP024581)
IS24887(SRP116943)
IS22632(SRP116972)
IS2205(SRP116981)
IS15401(SRP116992)
IS14317(SRP116941)
IS1054(M35-1)(SRP116995)
GrassI(SRP024580)
E36-1(SRP116991)
CSM63E(SRP117001)
B963676(SRP116931)
B35(SRP024579)
Chr07-genes
0 Mbp
10 Mbp
20 Mbp
30 Mbp
40 Mbp
50 Mbp
60 Mbp
70 Mbp
Max
Gene density
Min
Gene density

(H)

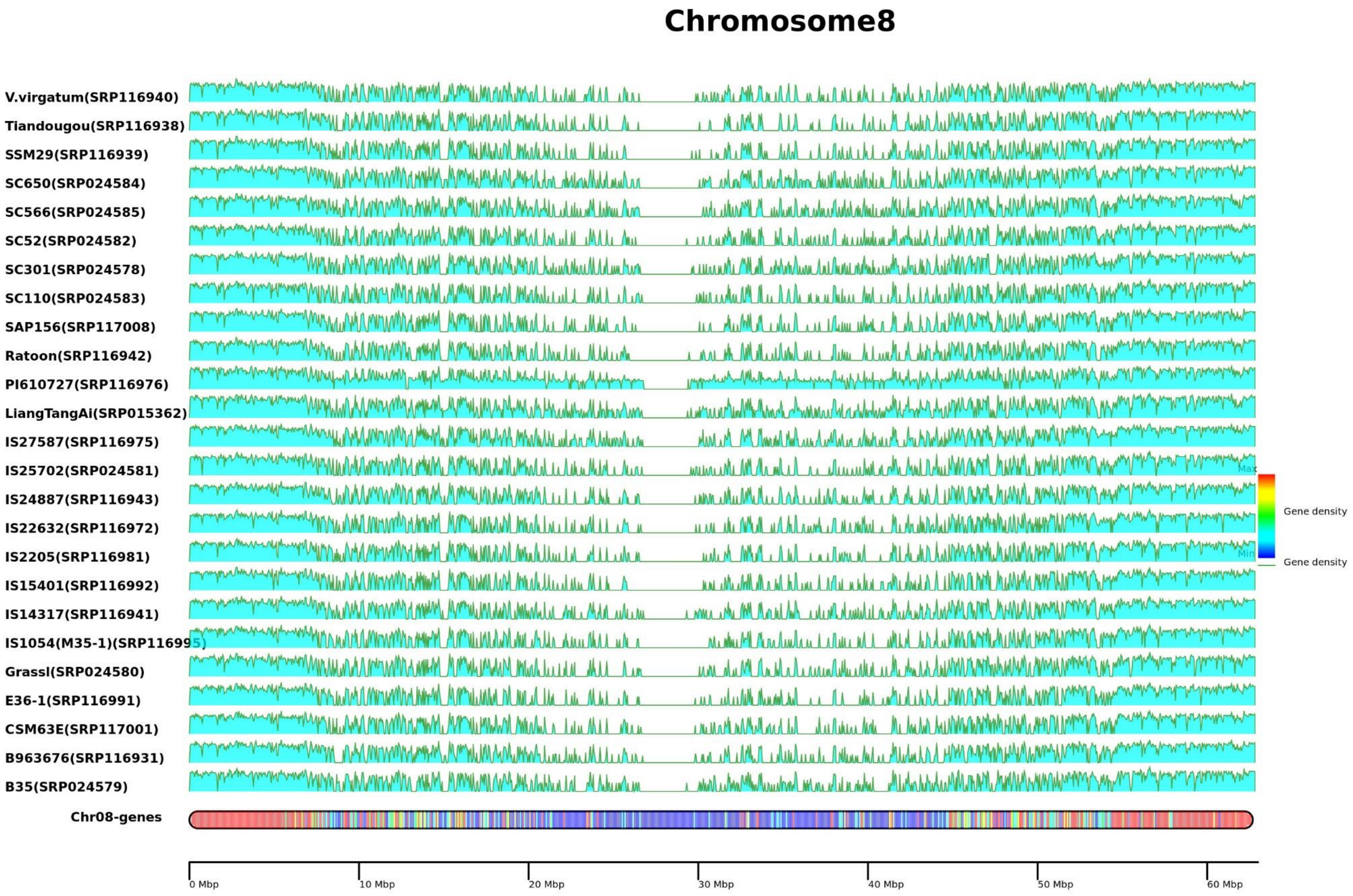
Chromosome8
V.virgatum(SRP116940)
Tiandougou(SRP116938)
SSM29(SRP116939)
SC650(SRP024584)
SC566(SRP024585)
SC52(SRP024582)
SC301(SRP024578)
SC110(SRP024583)
SAP156(SRP117008)
Ratoon(SRP116942)
PI610727(SRP116976)
LiangTangAi(SRP015362)
IS27587(SRP116975)
IS25702(SRP024581)
IS24887(SRP116943)
IS22632(SRP116972)
IS2205(SRP116981)
IS15401(SRP116992)
IS14317(SRP116941)
IS1054(M35-1)(SRP116995)
Grassl(SRP024580)
E36-1(SRP116991)
CSM63E(SRP117001)
B963676(SRP116931)
B35(SRP024579)
Chr08-genes
Max
Min
Gene density
Gene density
0 Mbp
10 Mbp
20 Mbp
30 Mbp
40 Mbp
50 Mbp
60 Mbp

(I)

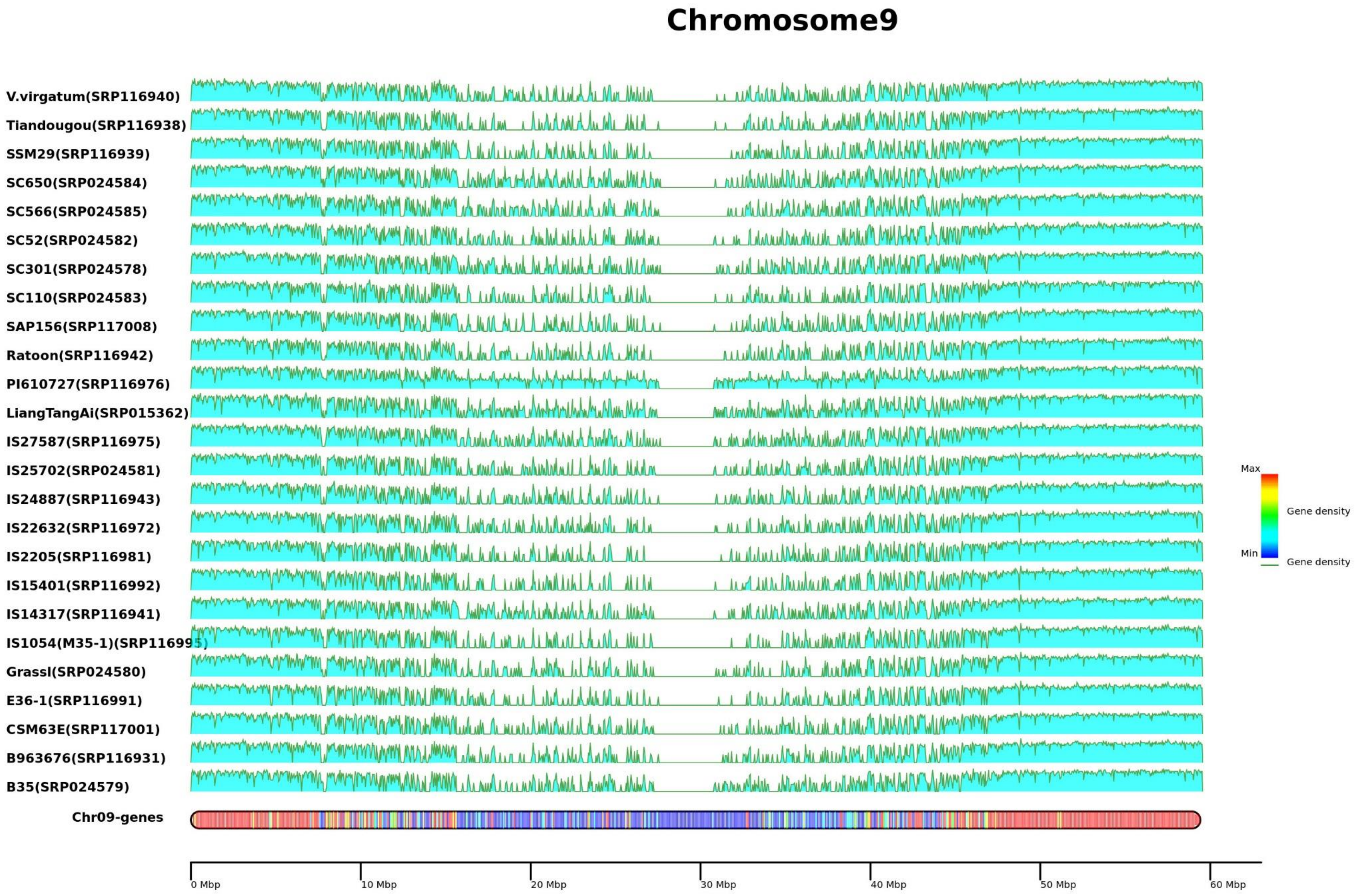
Chromosome9
V.virgatum(SRP116940)
Tiandougou(SRP116938)
SSM29(SRP116939)
SC650(SRP024584)
SC566(SRP024585)
SC52(SRP024582)
SC301(SRP024578)
SC110(SRP024583)
SAP156(SRP117008)
Ratoon(SRP116942)
PI610727(SRP116976)
LiangTangAi(SRP015362)
IS27587(SRP116975)
IS25702(SRP024581)
IS24887(SRP116943)
IS22632(SRP116972)
IS2205(SRP116981)
IS15401(SRP116992)
IS14317(SRP116941)
IS1054(M35-1)(SRP116995)
GrassI(SRP024580)
E36-1(SRP116991)
CSM63E(SRP117001)
B963676(SRP116931)
B35(SRP024579)
Chr09-genes
0 Mbp
10 Mbp
20 Mbp
30 Mbp
40 Mbp
50 Mbp
60 Mbp
Max
Min
Gene density
Gene density

(J)

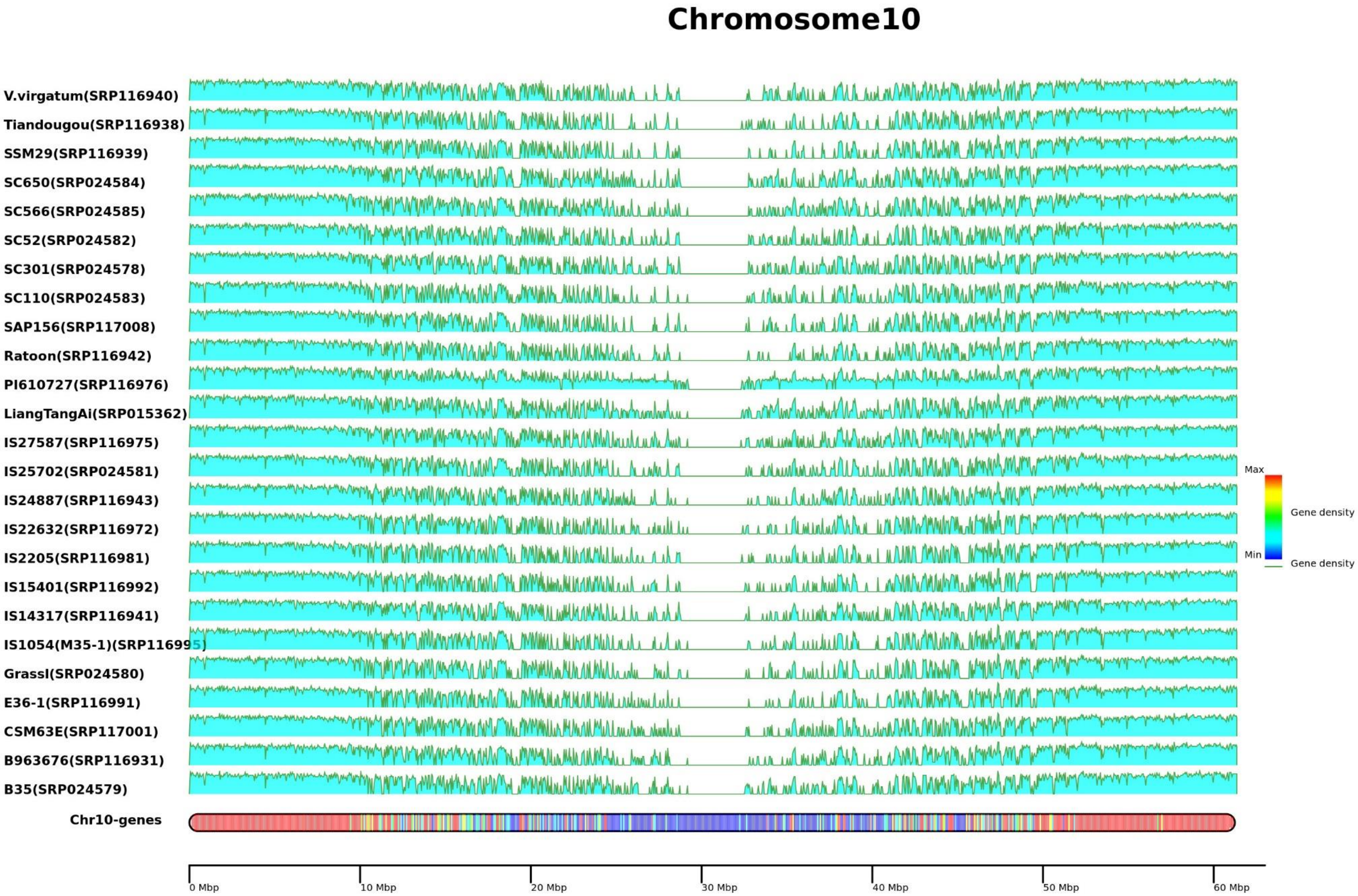
Chromosome10
V.virgatum(SRP116940)
Tiandougou(SRP116938)
SSM29(SRP116939)
SC650(SRP024584)
SC566(SRP024585)
SC52(SRP024582)
SC301(SRP024578)
SC110(SRP024583)
SAP156(SRP117008)
Ratoon(SRP116942)
PI610727(SRP116976)
LiangTangAi(SRP015362)
IS27587(SRP116975)
IS25702(SRP024581)
IS24887(SRP116943)
IS22632(SRP116972)
IS2205(SRP116981)
IS15401(SRP116992)
IS14317(SRP116941)
IS1054(M35-1)(SRP116995)
GrassI(SRP024580)
E36-1(SRP116991)
CSM63E(SRP117001)
B963676(SRP116931)
B35(SRP024579)
Chr10-genes
Max
Gene density
Min
Gene density
0 Mbp
10 Mbp
20 Mbp
30 Mbp
40 Mbp
50 Mbp
60 Mbp

(K)

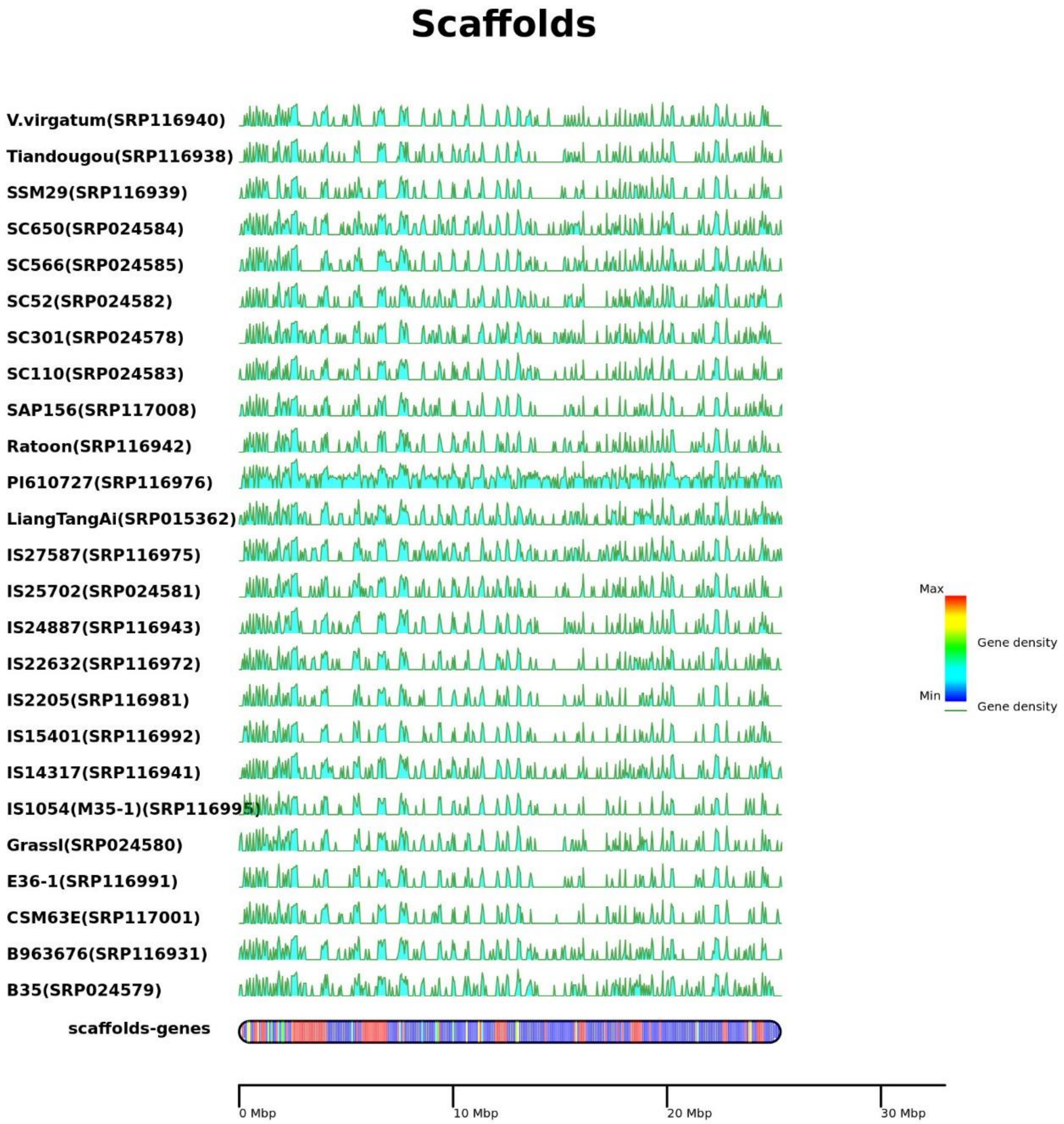
Scaffolds
V.virgatum(SRP116940)
Tiandougou(SRP116938)
SSM29(SRP116939)
SC650(SRP024584)
SC566(SRP024585)
SC52(SRP024582)
SC301(SRP024578)
SC110(SRP024583)
SAP156(SRP117008)
Ratoon(SRP116942)
PI610727(SRP116976)
LiangTangAi(SRP015362)
IS27587(SRP116975)
IS25702(SRP024581)
IS24887(SRP116943)
IS22632(SRP116972)
IS2205(SRP116981)
IS15401(SRP116992)
IS14317(SRP116941)
IS1054(M35-1)(SRP116995)
Grassl(SRP024580)
E36-1(SRP116991)
CSM63E(SRP117001)
B963676(SRP116931)
B35(SRP024579)
scaffolds-genes
0 Mbp
10 Mbp
20 Mbp
30 Mbp
Max
Gene density
Min
Gene density

(L)

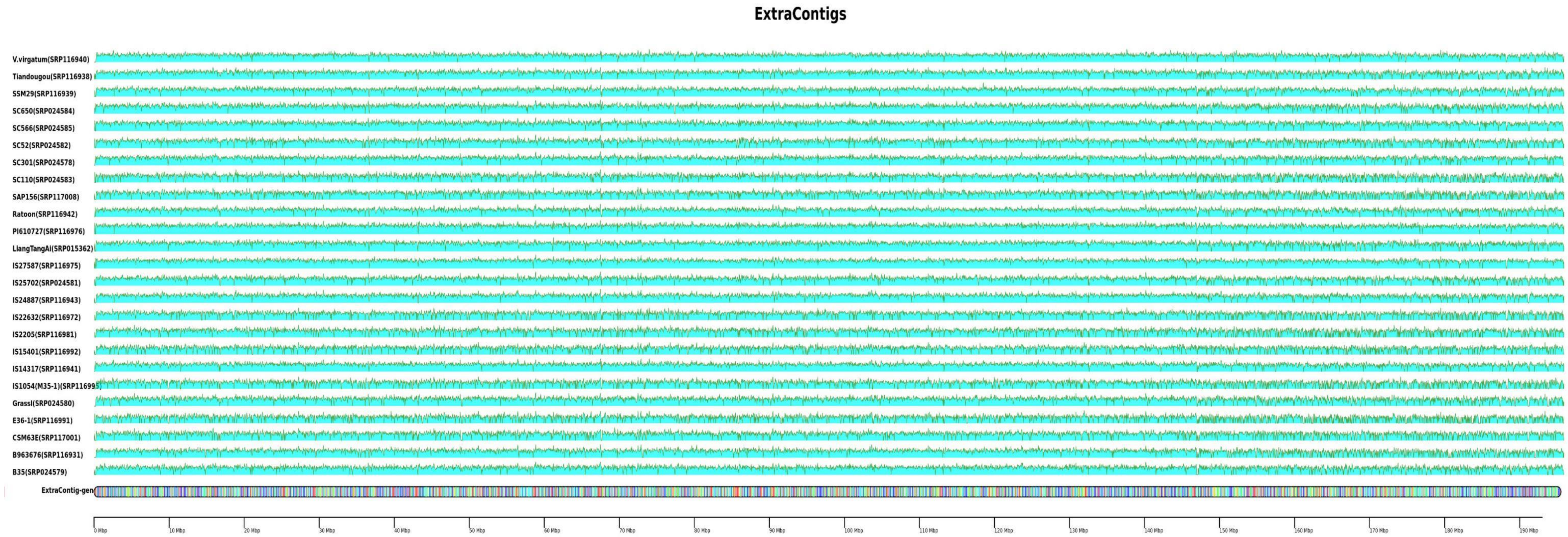

Supplementary Figure 5: Sorghum 25 accessions (names and NCBI accessions) RNASeq read mapping density on pan-genome assembly for **(A-J)** Chromosome 1-10 **(K)** Scaffold sequence put together as single sequence and **(L)** Non reference sequence assembly contigs from sorghum accessions concatenated to single sequence as extra contig sequence.
